# DAPL1 prevents epithelial–mesenchymal transition in the retinal pigment epithelium and experimental proliferative vitreoretinopathy

**DOI:** 10.1038/s41419-023-05693-4

**Published:** 2023-02-25

**Authors:** Xiaoyin Ma, Shuxian Han, Youjia Liu, Yu Chen, Pingping Li, Xiaoyan Liu, Lifu Chang, Ying-ao Chen, Feng Chen, Qiang Hou, Ling Hou

**Affiliations:** 1 Laboratory of Developmental Cell Biology and Disease, School of Ophthalmology and Optometry and Eye Hospital, Wenzhou Medical University, Wenzhou, 325003 China; 2 State Key Laboratory of Ophthalmology, Optometry and Vision Science, Wenzhou Medical University, Wenzhou, 325003 China; 3 Department of Ophthalmology, First Affiliated Hospital of Anhui Medical University, Hefei, 230022 China; 4 School of Ophthalmology and Optometry and Eye Hospital, Wenzhou Medical University, Wenzhou, 325003 China

**Keywords:** Experimental models of disease, Preclinical research

## Abstract

Epithelial–mesenchymal transition (EMT) of the retinal pigment epithelium (RPE) is a hallmark of the pathogenesis of proliferative vitreoretinopathy (PVR) that can lead to severe vision loss. Nevertheless, the precise regulatory mechanisms underlying the pathogenesis of PVR remain largely unknown. Here, we show that the expression of death-associated protein-like 1 (DAPL1) is downregulated in PVR membranes and that DAPL1 deficiency promotes EMT in RPE cells in mice. In fact, adeno-associated virus (AAV)-mediated DAPL1 overexpression in RPE cells of *Dapl1-*deficient mice inhibited EMT in physiological and retinal-detachment states. In a rabbit model of PVR, ARPE-19 cells overexpressing DAPL1 showed reduced ability to induce experimental PVR, and AAV-mediated DAPL1 delivery attenuated the severity of experimental PVR. Furthermore, a mechanistic study revealed that DAPL1 promotes P21 phosphorylation and its stabilization partially through NFκB (RelA) in RPE cells, whereas the knockdown of P21 led to neutralizing effects on DAPL1-dependent EMT inhibition and enhanced the severity of experimental PVR. These results suggest that DAPL1 acts as a novel suppressor of RPE-EMT and has an important role in antagonizing the pathogenesis of experimental PVR. Hence, this finding has implications for understanding the mechanism of and potential therapeutic applications for PVR.

## Introduction

Proliferative vitreoretinopathy (PVR) is a disease that arises as a complication of rhegmatogenous retinal detachment, is characterized by the formation of subretinal or epiretinal membranes, and can lead to vision loss or severe blindness [1]. PVR occurs in 5–10% of retinal detachment cases, and surgical treatment is the main current therapy, but recurrence occurs readily after the operation [2]. Retinal pigment epithelium (RPE) cells are the principal cell components within the PVR membranes, and epithelial–mesenchymal transition (EMT) in these cells is considered the hallmark of PVR pathogenesis [3, 4]. During PVR progression, the EMT of RPE cells can promote their reprogramming to fibrotic cells, leading to the formation of a contractile epiretinal or subretinal membrane [1, 5, 6]; however, the regulatory mechanisms underlying this progression remain largely unknown. It is therefore paramount to explore the molecular mechanisms driving this process during PVR in more detail to identify more specific therapeutic targets for future precision therapeutic interventions.

The mature RPE is a monolayer of tightly connected pigmented cells abutting retinal photoreceptors that plays critical roles in the normal retinal structure and visual function as it absorbs scattered light, maintains the blood-retinal barrier, is involved in the material transfer, secretes trophic factors, and clears shed photoreceptor outer segment fragments via phagocytosis [7, 8]. Under the disease conditions of retinal detachment or retinal surgery, RPE cells will migrate into the vitreous, undergo EMT, re-enter the cell cycle, and eventually develop into contractile membranes on retinal surfaces to cause PVR and subsequent visual impairment [9, 10]. During the EMT process, the morphology of RPE cells is changed, the expression of epithelial molecular markers, such as the tight junction zonula occludens protein 1 (ZO-1), is decreased, and the expression of mesenchymal molecular markers, such as α-smooth muscle actin (α-SMA) and vimentin, is increased [11, 12]. The tight junction and barrier function of RPE cells will thus be damaged after EMT, and inflammatory factors and external substances can then enter the retina to cause inflammation.

Previous investigations have shown that various growth factors and cytokines (such as TGF-β), intracellular signaling pathways (such as WNT), transcription factors (SNAIL, ZEB1), and other noncoding RNAs are involved in the regulation of EMT in RPE cells [4, 13, 14]. However, RPE-EMT is a complicated process, the precise underlying mechanisms remain largely unknown, and effective therapeutic or preventive strategies for clinical PVR have not yet become readily available. Therefore, there is an urgent need to further identify novel regulators involved in the EMT of RPE cells and elucidate their functional roles in PVR progression; these could be exploited to treat PVR by targeting RPE cells.

Death-associated protein-like 1 (DAPL1, also known as early epithelial differentiation-associated protein, EEDA) is expressed in epithelial cells [15], and its genetic variant is associated with age-related macular degeneration (AMD) [16]. DAPL1 could be involved in the differentiation of renal mesenchymal progenitor cells and the morphogenesis and development of ovarian granulosa cells [17–19]. Nevertheless, although DAPL1 highly expresses in the RPE and inhibits the proliferation of RPE cells [20, 21], its functional role in the inhibition of EMT in RPE cells is still unknown.

In the present study, we found that the expression level of *DAPL1* is decreased in the PVR membrane compared with that in primary human RPE cells. In addition, we show that knockout of the *Dapl1* gene in mice promotes the EMT of RPE cells both in physiological and retinal-detachment states in vivo, whereas AAV-p.RPE65-DAPL1 mediated gene transfer inhibits RPE-EMT in the *Dapl1*−*/−* mice. Furthermore, we reveal that the lentivirus-mediated overexpression of DAPL1 in ARPE-19 cells reduces their ability to induce experimental PVR in the Chinchilla rabbit and that AAV-CMV-DAPL1 gene delivery can antagonize the severity of experimental PVR progression. Mechanistically, DAPL1 was found to promote P21 phosphorylation and its stabilization by up-regulating NFκB (RelA) in RPE cells, whereas the knockdown of P21 in DAPL1-overexpressing ARPE-19 cells neutralized its functions in inhibiting EMT and promoted experimental PVR progression. Hence, it appears that the DAPL1-NFκB-P21 pathway in RPE cells is an important contributor to RPE cell homeostasis.

## Materials and methods

### Ethical approval

The current research involving clinical samples was approved by the ethics committee of Wenzhou Medical University Eye hospital (2020–007-K-07). All animal experiments were approved by the Wenzhou Medical University Animal Care and Use Committee and performed in compliance with the Association for Research in Vision and Ophthalmology (ARVO) Statement on the Use of Animals in Ophthalmic and Vision Research (WYDW2022-0193).

### Animals used in this study

CRISPR/Cas9-mediated *Dapl1*-knockout mice (*Dapl1*−/−) were generated in the Nanjing Biomedical Research Institute of Nanjing University, China, as described in our previous work [20, 22]. C57BL/6J mice were obtained from The Jackson Laboratory and were maintained in the specific pathogen-free facility of the Wenzhou Medical University, China. Pigmented rabbits (Chinchilla rabbits) were purchased from Danyang Changyi experimental animal breeding Co., Ltd, China, and cultured in the animal center of Wenzhou Medical University.

### Cell culture

The ARPE-19 cells were purchased from ATCC and cultured in DMEM/F12 supplemented with 10% FBS, 100 U/ml of penicillin, and 100 mg/ml of streptomycin. The DAPL1-overexpressing ARPE-19 cells (ARPE-19 + DAPL1) or EGFP-overexpressing EGFP cells (ARPE-19+EGFP) were produced as previously described [20]. P21-stable knockdown ARPE-19 + DAPL1 cells were established using lentivirus-mediated shP21 infection.

### Mouse retinal detachment and rabbit experimental PVR models

After being anesthetized with 10% pentobarbital sodium, 1 μl of 0.25% sodium hyaluronate was injected into the subretinal space of 8-week-old WT or *Dapl1*−/− mice to cause retinal detachment, which was analyzed by H&E staining or immunostaining 10 days later. Rabbit experimental PVR was established as previously reported [23, 24]. Briefly, the adult Chinchilla rabbits were anesthetized and intravitreally injected with 20 μl of 5 × 10^5^ ARPE-19 cells. The fundus photographs and OCT were used for the examination after 30 and 38 days. PVR was graded using Fastenberg’s scale as described previously [4] and as follows: stage 0, normal fundus; stage 1, the presence of epiretinal membrane (ERM); stage 2, ERM with focal retinal traction or an abnormal vessel appearance; stage 3, localized retinal detachment (RD); stage 4, extensive RD of at least two quadrants without complete RD; stage 5, complete RD. For the pathological analysis, the rabbits were euthanized via intravenous injection of sodium pentobarbital, and the eyes were enucleated. The eyes were fixed with 4% paraformaldehyde and embedded in paraffin. Sections (10 μm) were prepared and subjected to H&E staining and immunostaining for α-SMA.

### Immunofluorescence and quantification of fluorescent intensity

The immunofluorescence method was previously described [22]. For the quantification of the immunofluorescence intensity, a simple method for quantitating confocal fluorescent images was used [25]. Briefly, the relative fluorescence intensity of anti-α-SMA in each projected fluorescent image was performed using a Zeiss LSM880 confocal microscope with the fixed parameters between the experimental and control group using Image J. The images were color-split, the particle edges were smoothed, and each image intensity threshold was automatically adjusted and applied.

### AAV9 vector construction and virus injection

The AAV9-p.RPE65-MCS-SV40-PolyA and AAV9-CMV-MCS-SV40-PolyA vectors were acquired from Shanghai Genechem Co., LTD, China. Human HA-DAPL1 (NM_001017920) was constructed as previously described [21]. For the experiments in mice, 0.5 μl of AAV9-p.RPE65-DAPL1 or the AAV9 vector virus supernatant (10^12^ genome copies/ml) was injected into the subretinal spaces of mice, using a pulled angled glass pipette under direct observation aided by a dissecting microscope under dim light [22]. For the experiments in rabbits, 2 μl of AAV9-CMV-DAPL1 or the AAV9 vector virus supernatant (10^12^ genome copies/ml) was injected into the vitreous of the rabbits.

### Gene overexpression and knockdown

The lentivirus-mediated overexpression of DAPL1 in ARPE-19 cells was described in our previous work [20]. SiRNAs or shRNA were used to knockdown respective target genes. Lentivirus harboring shP21, with the sequence of aaGACCATGTGGACCTGTCAC (10^8^ genome copies/ml), was purchased from Shanghai Genechem Co., LTD, China. Specific siRNAs for human P21 and NFκB were synthesized by Gene Pharma Co. (Shanghai, China) with the following sequences: si-P21-1, CAGGCGGUUAUGAAAUUCATT; si-P21-2, GAUGGAACUUCGACUUUGUTT; si-P21-3, CCUCUGGCAUUAGAAUUAU; si-RelA-1, GCACCAUCAACUAUGAUGATT; si-RelA-2, GGAGUACCCUGAGGCUAUATT; si-RelA-3, UCUUCCUACUGUGUGACAATT; si-NC, AUUUCUUUCAUGUUGUGGGTT. For transfections, 5 μl of siRNA and 5 μl of Lipofectamine™ 2000 were diluted with 90 μl of serum-free F12/DMEM medium and mixed after 5 min incubation. The mixture was then incubated for 20 min at room temperature and added drop-wise to each culture well containing 800 μl of serum-free F12/DMEM medium (final siRNA concentration, 100 nM). The medium was discarded 6 h thereafter and replaced with fresh complete culture medium with 10% FBS. Experiments such as western blotting were performed after culturing for 48 h.

### Protein stability assay

CHX (cycloheximide, 10 μg/ml) was added to the culture medium of ARPE-19 + EGFP or ARPE-19 + DAPL1 cells, and cells were collected at different time points; proteasome inhibitor MG132 (20 μg/ml) was added to the culture medium of ARPE-19 + EGFP or ARPE-19 + DAPL1 cells for 4 h, the total lysates were analyzed by immunoblotting using an anti-P21antibody.

### Antibodies and primer sequences

Anti-DAPL1 (ab150969), anti-P-Cadherin (ab242060), anti-ZEB1 (ab87280) and anti-p-P21 (ab47300) were purchased from Abcam. Anti-ZO-1 (8193T), anti- E-Cadherin (3195S) anti-N-cadherin (13116T), anti-vimentin (5741T), anti-SNAIL (3879T), anti-αSMA (19245S), anti-P-smad2/3 (8828S), anti-P21 (2947S) and anti- NFκB (RelA) (4764s) were obtained from Cell Signaling Technology. The anti-GAPDH (KC-5G4) antibody was purchased from Aksomics. The anti-OTX2 (AF1979) antibody was purchased from R&D systems. The anti-p-P38 MAPK (AM063) antibody was purchased from Beyotime China. Primer sequences for real-time PCR were as follows: DAPL1-F, GAAAGCTGGAGGGATGCGAA; DAPL1-R, TGATGTCCGTGTGAACTGT; GAPDH-F, AGGTCGGTGTGAACGGATTTG; GAPDH-R, TGTAGACCATGTAGTTGAGGTCA; P21-F, AGTCAGTTCCTTGTGGAGCC; P21-R, CATTAGCGCATCACAGTCGC.

### Statistical analysis

Each cell-based experiment was repeated three times, whereas mouse and rabbit experiments were repeated six times. All quantitative data were presented as the mean ± SEM. The statistical significance of differences between groups was obtained using one-way ANOVA with GraphPad (San Diego, CA). Differences were considered significant at *P* < 0.05.

## Results

### *Dapl1* deficiency promotes the EMT of RPE cells

**Fig. 1:**
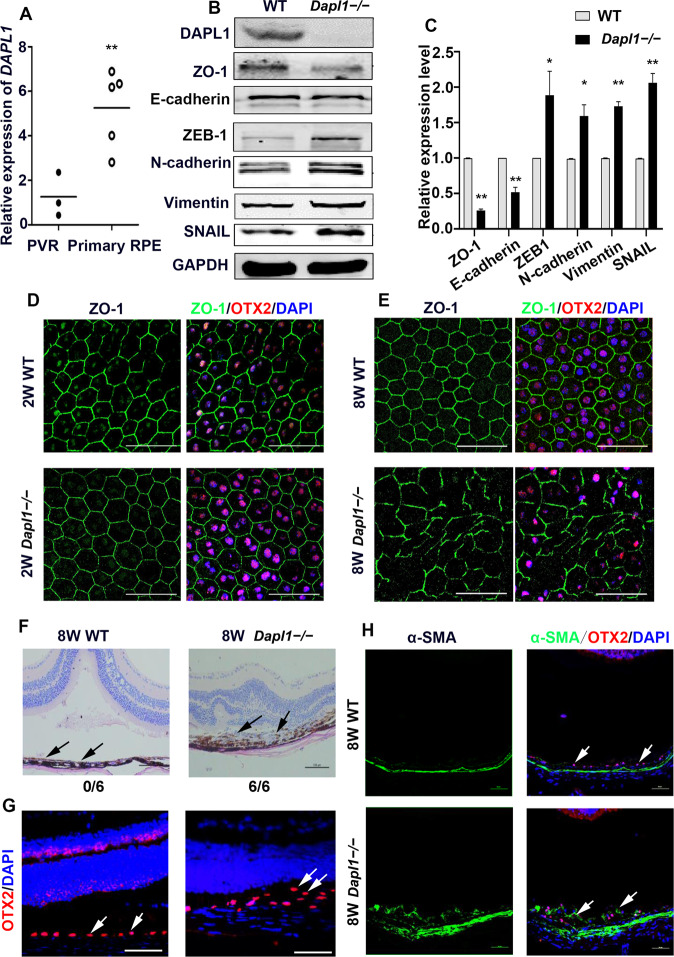
DAPL1 deficiency promotes the EMT of RPE cells. **A** Real-time PCR showing the expression level of DAPL1 in proliferative vitreoretinopathy (PVR) membranes and primary human RPE cells. **B** Western blotting showing the protein expression levels of DAPL1, ZO-1, E-cadherin, ZEB1, N-cadherin, vimentin, and SNAIL in 8-week-old WT and *Dapl1* − /− RPE cells. **C** The bar graph shows quantification of the bands based on the results in (**B**). **D**, **E** Immunostaining images of ZO-1 expression in RPE flat mounts of 2-week-old or 8-week-old WT and *Dapl1*−/− mice. Scale bar: 50 μm. **F** Histological images of H&E staining of the retinal structure showing that 8-week-old WT and *Dapl1*−/− mice were subretinally injected with sodium hyaluronate to induce retinal detachment for 10 days Scale bar: 100 μm. (**G**, **H**) Immunohistochemistry images showing the expression of OTX2 (white arrows in **G**) and α-SMA (white arrows in H) on the surfaces of the detached retinas. Scale bar: 50 μm. *N* = 6, ** or * indicates *P* < 0.01 or *P < 0.05, respectively.

To address whether DAPL1 plays any role in the EMT of RPE cells and PVR progression, the expression level of DAPL1 in the PVR membrane was analyzed and compared with that in primary human RPE cells. As shown in Fig. 1A, the expression of *DAPL1* in the PVR membrane was significantly lower than that in the primary human RPE cells, suggesting that DAPL1 might be involved in the regulation of an RPE-EMT state and PVR progression. To further address this question, *Dapl1*-deficient mice (hereafter named *Dapl1*−/−) were used to analyze EMT in RPE cells. Western blot results showed that RPE cells in *Dapl1*−/− mice did not express DAPL1 protein, whereas expression of the epithelial marker ZO-1 and E-cadherin were decreased and that of the mesenchymal biomarkers ZEB1, N-cadherin, vimentin, and SNAIL were significantly increased (Fig. 1B, C). To further confirm the functional roles of DAPL1 in RPE-EMT progression, ZO-1 immunostaining was performed on RPE flat mounts of 2-week- and 8-week-old wild-type (hereafter WT) and *Dapl1*−/− mice. As shown in Fig. 1D, RPE cells displayed a regular hexagonal structure in 2-week-old WT and *Dapl1*−/− mice (Fig. 1D). Interestingly, compared with the regular hexagonal structure of RPE cells in 8-week-old WT mice, the tight connections among RPE cells were lost and the cell morphology was partially changed to a mesenchymal phenotype in 8-week-old *Dapl1*−/− mice (Fig. 1E). These results suggest that DAPL1 deficiency promotes RPE-EMT under normal physiological conditions.

As retinal detachment is one of the causative factors of PVR, we next examined whether DAPL1 deficiency would promote experimental PVR progression. For this, we performed subretinal injection of sodium hyaluronate to induce retinal detachment in 8-week-old WT and *Dapl1*−/− mice and maintained them for 10 days. As shown in Fig. 1F, RPE cells remained in a single-layer state in the WT mice, but they migrated into the detached subretinal space, underwent mesenchymal transformation, and proliferated abnormally in the *Dapl1*−/− mice, as shown by hematoxylin and eosin (H&E) staining (Fig. 1F, black arrow). In addition, those subretinal pigmented cells were positive for OTX2, indicating they were RPE cells (Fig. 1G, white arrow). We further analyzed expression of the mesenchymal marker α-SMA in the detached retina, and immunohistochemical staining showed that RPE cells expressed low levels of this marker in WT mice, but its expression was dramatically increased in RPE cells of *Dapl1* − /− mice in the retinal-detachment state (Fig. 1H, white arrows, Fig. S1A). These data indicate that DAPL1 deficiency promotes the EMT of RPE cells in both physiological and retinal-detachment conditions.

### Overexpression of DAPL1 in the RPE inhibits EMT in *Dapl1*−*/−* mice

**Fig. 2:**
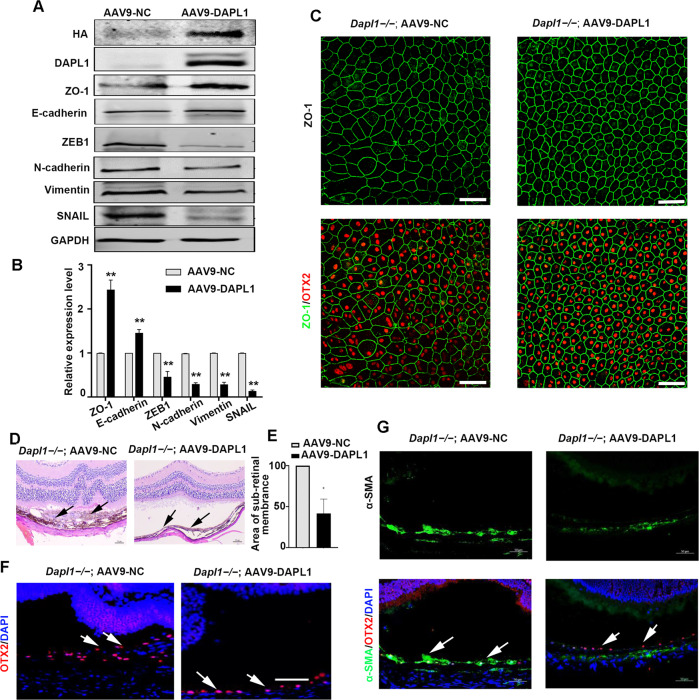
Overexpression of DAPL1 in the RPE of *Dapl1*−*/−* mice inhibits EMT in vivo. **A** At 2 weeks after infection with AAV9-NC or AAV9-DAPL1, western blotting showed the protein expression levels of HA tag, DAPL1, ZO-1, E-cadherin, ZEB1, N-cadherin, vimentin, and SNAIL in RPE cells of *Dapl1*−/− mice. **B** The bar graph shows quantification of the bands based on the results in (**A**). *N* = 3. **C** At 2 weeks after infection with AAV9 or AAV9-DAPL1, immunostaining images of ZO-1 in RPE flat mounts of 6-week-old *Dapl1*−/− mice. **D** At 2 weeks after the injection of AAV9-NC or AAV9-DAPL1, with the subsequent subretinal injection of sodium hyaluronate to cause retinal detachment for 10 days, histological images of H&E staining show retinal structures from 6-week-old *Dapl1*−/− mice. Note that subretinal membranes were observed in the control AAV9-NC- but not in the AAV9-DAPL1-injected eyes (black arrows). **E** The bar graph shows quantification of the area of the subretinal membrane based on the results from (**D**). **F** Immunohistochemistry for OTX2 (white arrow indicated) on the surfaces of the detached retinas of *Dapl1*−/− mice after the injection of AAV9-NC or AAV9-DAPL1 for 2 weeks. **G** Immunohistochemistry images showing α-SMA expression (white arrow indicated) on the surfaces of the detached retinas of *Dapl1*−/− mice after the injection of AAV9 or AAV9-DAPL1 for 2 weeks. *N* = 6, ** indicates *P* < 0.01. Scale bar: 50 μm.

As the aforementioned results indicated a role for DAPL1 in the EMT of RPE cells, we asked whether DAPL1 would play a protective role in this process. Hence, we overexpressed DAPL1 through AAV (adeno-associated virus)-mediated gene transfer using the RPE cell-specific *RPE65* (retinoid isomerohydrolase RPE65) promoter. As previously described, this promoter controls gene expression specifically in RPE cells [22], and we constructed the AAV9 expression vector AAV9-*p.RPE65*-*DAPL1-HA* for the expression of HA epitope-tagged DAPL1 and packaged it (hereafter called AAV9-DAPL1). To assess the functions of DAPL1 in RPE cells in vivo, AAV9-DAPL1 was injected into the subretinal space of the right eyes of 6-week-old *Dapl1*−/− mice, whereas AAV9-NC was injected into the left eyes as a control. Two weeks after virus injection, protein levels of DAPL1, HA, and EMT markers were analyzed by western blotting. As shown in Fig. 2A, B, AAV9-DAPL1 injection led to increased levels of HA-tagged DAPL1, promoted the expression of ZO-1, E-cadherin, and decreased the protein levels of ZEB1, N-cadherin, vimentin, and SNAIL in RPE cells of *Dapl1*−/− mice. Moreover, the overexpression of DAPL1 in RPE cells of *Dapl1*−/− mice restored the tight connections among and the hexagonal structure of RPE cells (Fig. 2C). Most importantly, mesenchymal transformed and abnormally proliferating RPE cells could be observed in the detached retina of the AAV9-NC injected eyes, but the area of the subretinal membrane was dramatically decreased in the AAV9-DAPL1-injected eyes (Fig. 2D, black arrow, 2E). OTX2 immunostaining further confirmed the multiple layers of RPE cells in the AAV9-injected eyes, whereas a single layer was observed in the AAV-DAPL1-injected eyes (Fig. 2F, white arrow). In addition, immunohistochemical staining showed that the expression of α-SMA was dramatically decreased in the detached retinas of the AAV-DAPL1-injected eyes (Fig. 2G, white arrow, Fig. S1B). These data suggest that the overexpression of DAPL1 in RPE cells can inhibit EMT in vivo.

### Overexpression of DAPL1 inhibits the EMT of RPE cells in vitro

**Fig. 3:**
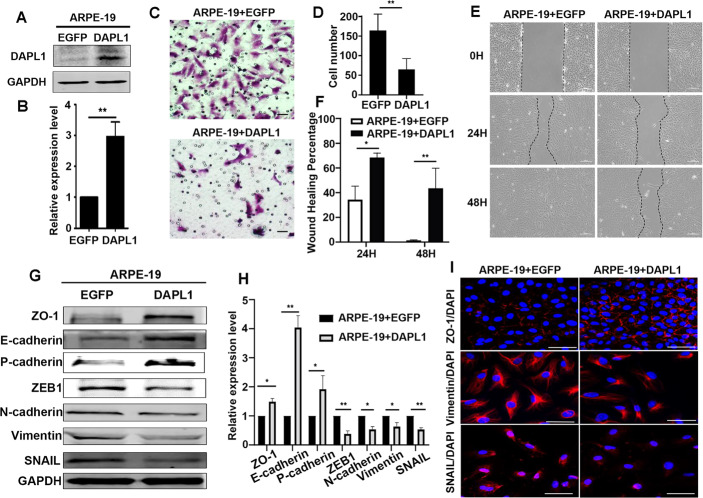
Overexpression of DAPL1 inhibits the EMT of RPE cells in vitro. **A**, **B** Western blotting showing the protein expression level of DAPL1 in ARPE-19 cells and its quantification. **C** Transwell assays for analyses of the migration of ARPE-19+EGFP or ARPE-19 + DAPL1 cells. **D** Quantification of the number of migrated cells based on the results from **C**. **E**, **F** Wound healing assay at 0, 24 and 48 h respectively in ARPE-19 + EGFP and ARPE-19 + DAPL1 cells and the wound healing percentage. Note that the cell migration was strongly inhibited by DAPL1 overexpression. **G**, **H** Western blotting showing the protein expression levels of ZO-1, E-cadherin, P-cadherin, ZEB1, N-cadherin, Vimentin, and SNAIL in ARPE-19+EGFP or ARPE-19 + DAPL1 cells and quantification. **I** The immunostaining images of ZO-1, Vimentin, and SNAIL in ARPE-19+EGFP or ARPE-19 + DAPL1 cells. *N* = 3, ** or * indicates *P* < 0.01 or *P < 0.05, respectively. Scale bar: 50 μm.

To further address the functional roles of DAPL1 in RPE-EMT and experimental PVR progression, we utilized in vitro experiments based on the overexpression of DAPL1 in a human RPE cell line, ARPE-19. We first elevated the expression level of DAPL1 via lentivirus-mediated DAPL1 overexpression (Fig. 3A, B). Transwell assay showed that the migration capacity of ARPE-19 cells decreased after the overexpression of DAPL1 (Fig. 3C, D). Meanwhile, wound healing assay showed that 48 hours after the wound was made, EGFP infected ARPE-19 cells almost covered the wound area, but the DAPL1 infected ARPE-19 cells migrated slower, with about 40% of the wounds are not healed (Fig. 3E, F). These results suggest that DAPL1 inhibits RPE-EMT. Next, we examined biomarkers of EMT in ARPE-19 cells by western blotting and immunostaining. As shown in Fig. 3G, H, the overexpression of DAPL1 in ARPE-19 cells increased the expression of ZO-1, E-cadherin, P-cadherin and decreased the expression of ZEB1, N-cadherin, vimentin and SNAIL, which was also confirmed by immunostaining (Fig. 3I). These results reveal that the overexpression of DAPL1 inhibits the EMT of ARPE-19 cells in vitro.

### DAPL1 inhibits the severity of experimental PVR progression

**Fig. 4:**
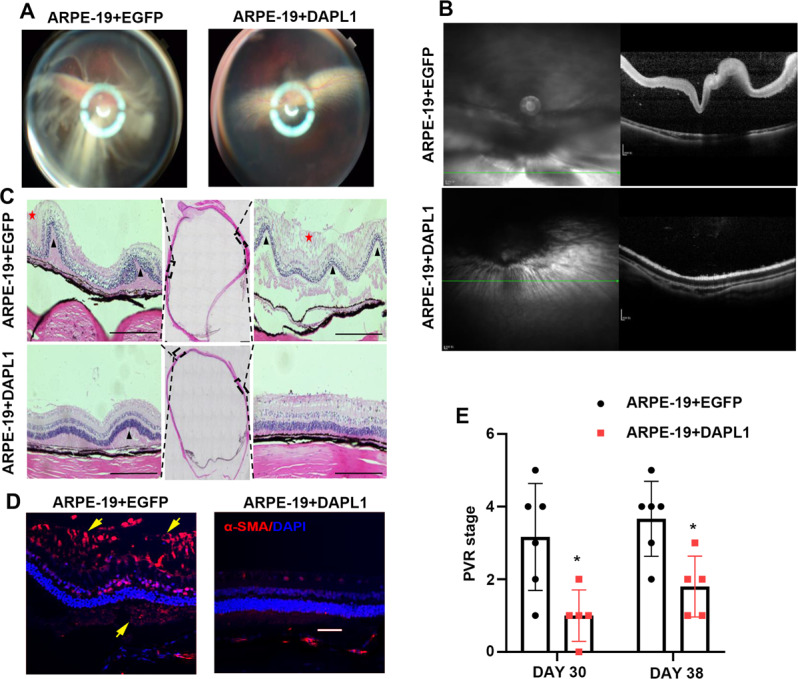
DAPL1 inhibits PVR progression in a rabbit model. ARPE-19+EGFP or ARPE-19 + DAPL1 cells were injected into the vitreous body of pigmented rabbits to induce experimental PVR. **A** Representative images showing fundus photographs captured on day 38 in each group. Note that severe retinal detachment with retinal folds was observed in ARPE-19+EGFP-injected eyes but not in ARPE-19 + DAPL1-injected eyes. **B** Optical coherence tomography (OCT) scanning showing that severe retinal detachment could be observed in the ARPE-19+EGFP-injected eyes but not in the ARPE-19 + DAPL1-injected eyes after 38 days. **C** Representative histological images of H&E staining of the rabbit eye in each group were obtained and analyzed. Note that retinal folds (black triangles), retinal detachment, and the formation of the epiretinal and subretinal membranes (red stars) were observed in the ARPE-19+EGFP-injected eyes but not in the ARPE-19 + DAPL1-injected eyes. **D** Immunohistochemistry images showing the detection of α-SMA expression around the retinas in the rabbits; positive signals were observed in the epiretinal and subretinal membranes in ARPE-19+EGFP-injected eyes (yellow arrows). **E** The severity of PVR in each group was graded according to Fastenberg’s score at the indicated times. *N* = 6, Scale bar: 50 μm.

Whereas the aforementioned results demonstrated that DAPL1 inhibits EMT in RPE cells, the relevant function of DAPL1 in PVR progression in vivo was still unclear. To determine whether DAPL1 exerts a suppressive effect on PVR, we used an experimental PVR rabbit model. To achieve this, 5 × 10^5^ ARPE-19+EGFP or ARPE-19 + DAPL1 cells in a 20 μl volume were injected into the vitreous of the Chinchilla rabbit, and PVR severity was evaluated after 30 and 38 days. As shown in Fig. 4A, B, based on fundus photographs and optical coherence tomography (OCT) analyses, ARPE-19+EGFP-injected eyes displayed severe retinal detachment, whereas the ARPE-19 + DAPL1-injected eyes had a much more organized retinal structure. H&E staining results also confirmed the results of the retinal detachment state (Fig. 4C). Furthermore, immunohistochemical staining showed that DAPL1 overexpression in ARPE-19 cells inhibited the formation of the epiretinal and subretinal membranes (Fig. 4C, red stars) and suppressed α-SMA expression (Fig. 4D, Fig. S1C). Finally, upon evaluating the severity of PVR according to Fastenberg’s score, the ability of ARPE-19 cells overexpressing DAPL1 to induce PVR was reduced when compared with that of ARPE-19 + EGFP cells (Fig. 4E). These results clearly indicate that DAPL1 inhibits the severity of experimental PVR progression.

### DAPL1 regulates P21 to inhibit the EMT of RPE cells in vitro

**Fig. 5:**
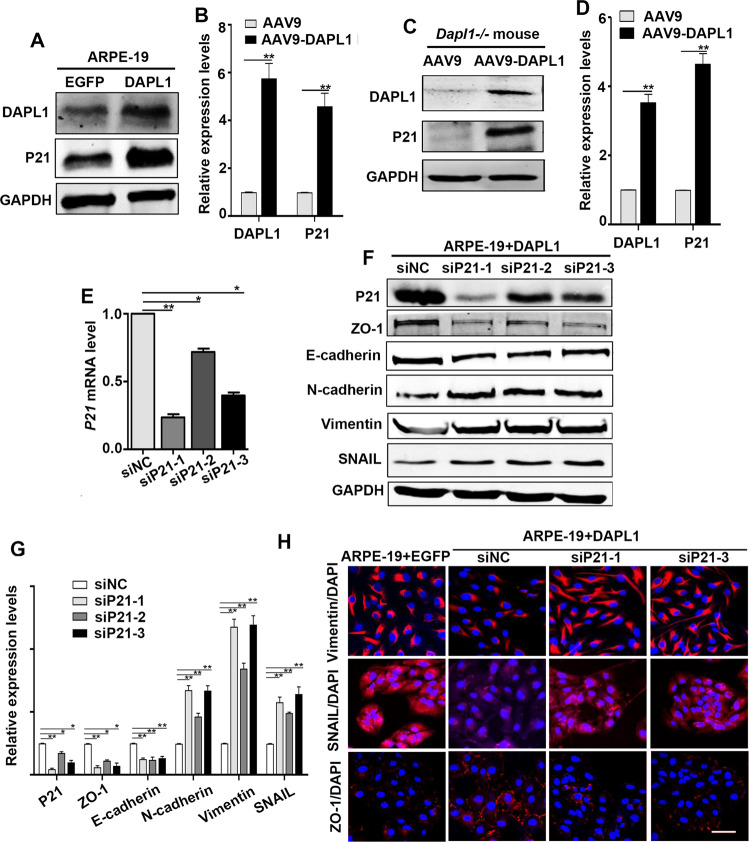
DAPL1 inhibits the EMT of RPE cells by regulating P21. **A**, **B** Western blots showing the expression of DAPL1 and P21 in ARPE-19 + DAPL1 cells and quantification (B). **C, D** At 2 weeks after infection with AAV9 or AAV9-DAPL1, western blots show the expression of DAPL1 and P21 in *Dapl1* − /− mouse RPE cells and quantification (**D**). **E** Real-time PCR showing the knockdown efficiency of P21 in ARPE-19 + DAPL1 cells. **F**, **G** Western blots showing the expression of P21, ZO-1, E-cadherin, N-cadherin, Vimentin, and SNAIL in ARPE-19 + DAPL1 cells after the knockdown of P21 and quantification. **H** Immunostaining images for ZO-1, vimentin, and SNAIL in ARPE-19 + DAPL1 cells after the knockdown of P21. *N* = 3, ** or * indicates *P* < 0.01 or *P < 0.05, respectively.

P21 is a cell proliferation inhibitor that plays an important regulatory role in the proliferation and differentiation of RPE cells [26]. Our previous work has demonstrated that DAPL1 upregulates the protein level of P21 in RPE cells, but the underlying mechanisms are unclear [20]. The overexpression of P21 in RPE cells can inhibit the progression of PVR in the rabbit model [27]. The P21 level increased in studies showing the inhibitory effects of crocetin or trichostatin A on the RPE-EMT [28, 29]. These facts prompted us to analyze whether DAPL1 regulates the EMT of RPE cells through P21. We showed that DAPL1-overexpressing ARPE19 cells expressed higher protein levels of P21 (Fig. 5A, B), and the AAV9-DAPL1-mediated overexpression of DAPL1 in *Dapl1*−/− mouse RPE cells dramatically elevated the protein level of P21 (Fig. 5C, D). Then, we addressed whether the knockdown of P21 in ARPE-19 + DAPL1 cells could neutralize its functional role in inhibiting EMT in RPE cells. As shown in Fig. 5E, siRNAs specifically targeting P21 were able to significantly knock down the expression of P21 in ARPE-19 + DAPL1 cells. The protein level of the epithelial cell markers ZO-1 and E-cadherin were decreased, whereas the EMT markers N-cadherin, vimentin and SNAIL were increased in the ARPE-19 + DAPL1 cells after P21 knockdown (Fig. 5F, G), which was also confirmed by the immunostaining (Fig. 5H). These results suggest that DAPL1 regulates the EMT of RPE cells at least in part through P21.

### DAPL1 increases P21 stability through NFκB

**Fig. 6:**
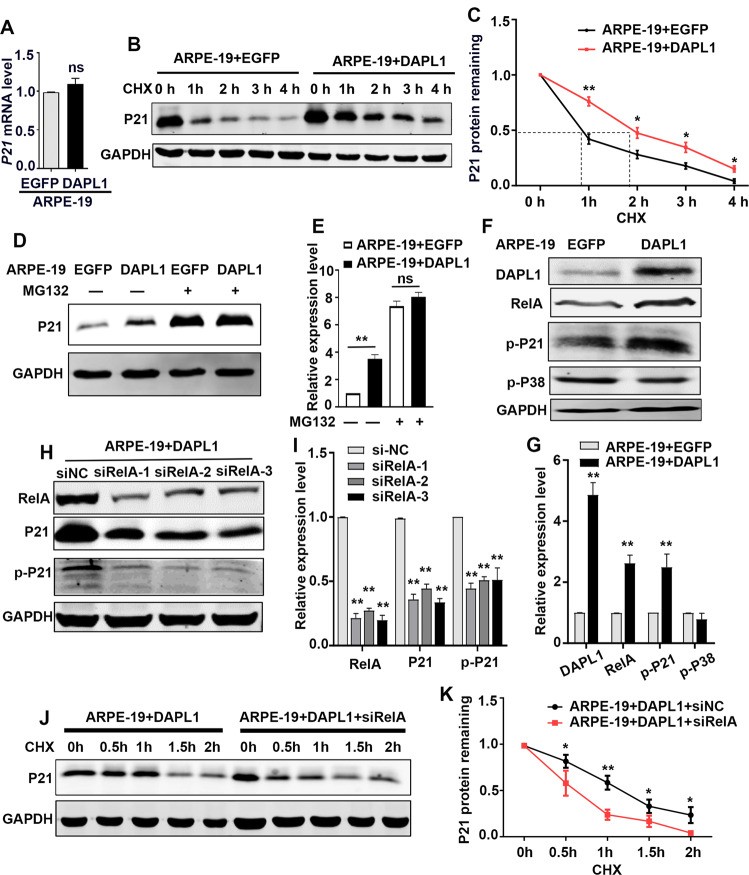
DAPL1 promotes P21 stability through NFκB. **A** Real-time PCR showing the mRNA expression level of *P21* in ARPE-19 + DAPL1 cells. **B** Western blots showing the level of P21 protein in ARPE-19+EGFP or ARPE-19 + DAPL1 cells upon treatment with cycloheximide (CHX) for various times, as indicated. **C** Line chart showing the percentage of remaining P21 protein at different times based on the results from (**B**). (**D**, **E**) Western blots showing the protein level of P21 in ARPE-19+EGFP or ARPE-19 + DAPL1 cells upon treatment with proteasome inhibitor MG132 for 4 h or not, as indicated. **F**, **G** Western blots showing the expression of DAPL1, NFκB, p-P21 and p-P38 in ARPE-19 + DAPL1 cells and quantification of the bands. **H**, **I** Western blots showing the expression of NFκB, P21 and p-P21 in ARPE-19 + DAPL1 cells, where NFκB was knocked down, and quantification results. **J** After the knockdown of NFκB in ARPE-19-DAPL1 cells for 48 h and subsequent treatment with CHX for various times, as indicated, the expression level of P21 protein was analyzed by western blotting. **K** The line chart shows the percentage of remaining P21 protein at different times based on (**J**). *N* = 3, ** indicates *P* < 0.01 or *P < 0.05, respectively.

The aforementioned data demonstrated that DAPL1 promotes an increase in the protein level of P21 and inhibits RPE-EMT; however, how DAPL1 regulates P21 in RPE cells was still unclear. Hence, to analyze the mechanism by which DAPL1 leads to the increase in P21 in RPE cells, we focused on whether DAPL1 regulates P21 expression. We first analyzed the mRNA expression of *P21* by real-time PCR and showed there was no significant difference between ARPE-19+EGFP and ARPE-19 + DAPL1 cells (Fig. 6A), suggesting that DAPL1 does not affect P21 at the transcriptional level, further prompting us to investigate whether DAPL1 affects P21 protein levels through post-transcriptional regulation, such as by modulating the stability of P21 protein. Hence, ARPE-19+EGFP or ARPE-19 + DAPL1 cells were treated with a translation inhibitor, cycloheximide (CHX) for 1 to 4 h. As expected, P21 protein had a prolonged half-life and was relatively abundant in the ARPE-19 + DAPL1 cells (Fig. 6B, C). Intriguingly, ARPE-19+EGFP and ARPE-19 + DAPL1 cells express similar protein levels of P21 after the treatment with proteasome inhibitor MG132 for 4 h (Fig. 6D, E). These results suggest that DAPL1 promotes the stability of the P21 protein in RPE cells. The P21 phosphorylation regulates its stability by suppressing proteasomal degradation [30]. Consistently, our data showed that DAPL1 increased the phosphorylation level of P21 (p-P21) in RPE cells; but did not affect the p-P38 protein level (Fig. 6F, G). Based on the fact that DAPL1 was not found to affect AKT and ERK pathways [20], we hypothesized that DAPL1 regulates P21 phosphorylation and stabilization through other regulatory mechanisms. It has been shown that NFκB (RelA) can positively regulate the protein level of P21 and stabilize P27 protein [31, 32], although it is unclear if NFκB (RelA) could be involved in the regulation of P21 stabilization. As shown in Fig. 6F, G, the protein level of NFκB (RelA) was increased when DAPL1 was overexpressed in ARPE-19 cells, whereas the knockdown of NFκB (RelA) in ARPE-19 + DAPL1 cells led to a decrease in the protein level of P21 (Fig. 6H, I). Moreover, the knockdown of NFκB (RelA) in ARPE-19 + DAPL1 cells for 2 days with subsequent CHX treatment for 0.5–2 h led to the increased degradation of P21 relative to that in the si-NC transfected cells (Fig. 6J, K). Taken together, these results indicate that DAPL1 promotes P21 phosphorylation and its stability at least partially through NFκB (RelA).

### P21 knockdown suppresses DAPL1-dependent experimental PVR inhibition

**Fig. 7:**
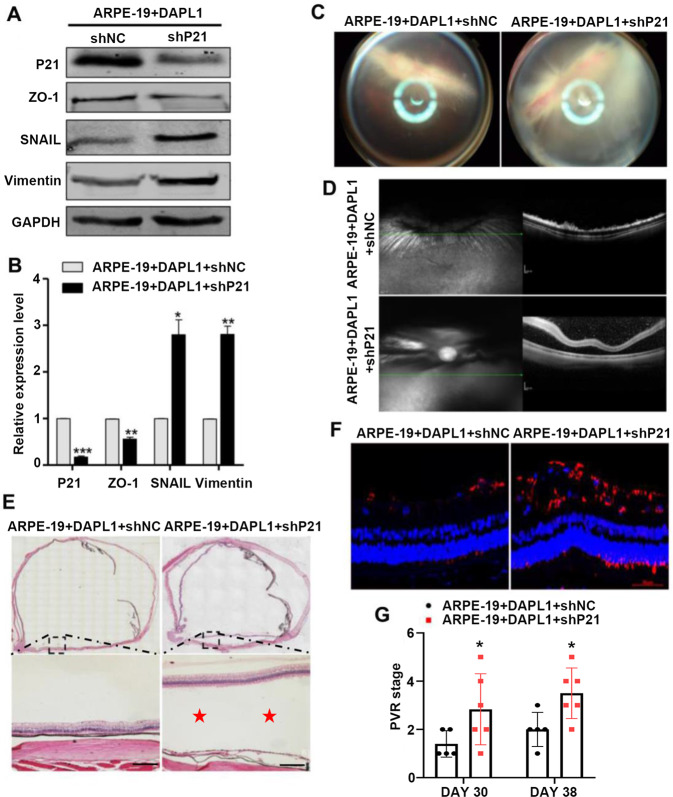
Knockdown of P21 in ARPE-19-overexpressing DAPL1 cells promotes PVR progression in a rabbit model. **A**, **B** Western blots showing the expression of P21, ZO-1, vimentin, and SNAIL in ARPE-19 + DAPL1 cells after the knockdown of P21 via infection with sh-P21 lentivirus and related quantification. **C** Representative images showing fundus photographs captured at day 38. Note that severe retinal detachment with retinal folds was observed in ARPE-19 + DAPL1 + sh-P21-injected eyes. **D** Optical coherence tomography (OCT) scans were performed on day 38. Note that severe retinal detachment was observed in the ARPE-19 + DAPL1 + shP21-injected eyes. **E** Histological images of H&E staining of the rabbit eye in each indicated group showing severe retinal detachment (red stars) in the ARPE-19 + DAPL1 + sh-P21-injected eyes. **F** Images show that α-SMA expression was detected by immunohistochemistry around the retinas in the rabbits, and its positive signals could be observed in the epiretinal and subretinal membranes in ARPE-19 + DAPL1 + shP21-injected eyes. **G** PVR severity in each group was graded according to Fastenberg’s score at the indicated times. *N* = 6, ** or * indicates *P* < 0.01 or *P < 0.05, respectively.

To determine whether P21 mediated the effect of DAPL1 in inhibiting PVR progression, we constructed an shRNA lentivirus targeting P21 (lv-sh-P21) and knocked down P21 in ARPE-19 + DAPL1 cells. As shown in Fig. 7A, B, the knockdown of P21 in ARPE-19 + DAPL1 cells led to a decrease in the expression of ZO-1 and an increase in levels of the EMT biomarkers vimentin and SNAIL. Then, we injected 5 × 10^5^ ARPE-19 + DAPL1 + shNC or ARPE-19 + DAPL1 + shP21 cells in a 20 μl volume into the vitreous of the Chinchilla rabbit, and PVR severity was evaluated after 30 and 38 days. After the injection of ARPE-19 + DAPL1 + shP21 cells into the eye, fundus photographs (Fig. 7C) and OCT (Fig. 7D) analyses showed that the injected eyes displayed severe retinal detachment, whereas the eyes injected with ARPE-19 + DAPL1 + shNC cells showed a much more organized retinal structure. H&E staining also confirmed the results of retinal detachment (Fig. 7E). Moreover, the knockdown of P21 in ARPE-19 + DAPL1 cells promoted the formation of epiretinal and subretinal membranes and resulted in increased expression of α-SMA in the membranes, as measured by immunohistochemical staining (Fig. 7F, Fig. S1D). In contrast, α-SMA expression was less obvious in the epiretinal and subretinal membranes after the injection of ARPE-19 + DAPL1 + shNC cells. Finally, upon evaluating PVR severity according to Fastenberg’s score, the ability of DAPL1-overexpressing ARPE-19 cells to induce PVR was increased after the knockdown of P21 at days 30 and 38 (Fig. 7G). These results clearly indicate that the knockdown of P21 attenuates the inhibitory effect of DAPL1 on the progression of experimental PVR.

### Gene transfer-mediated DAPL1 overexpression prevents the progression of PVR

**Fig. 8:**
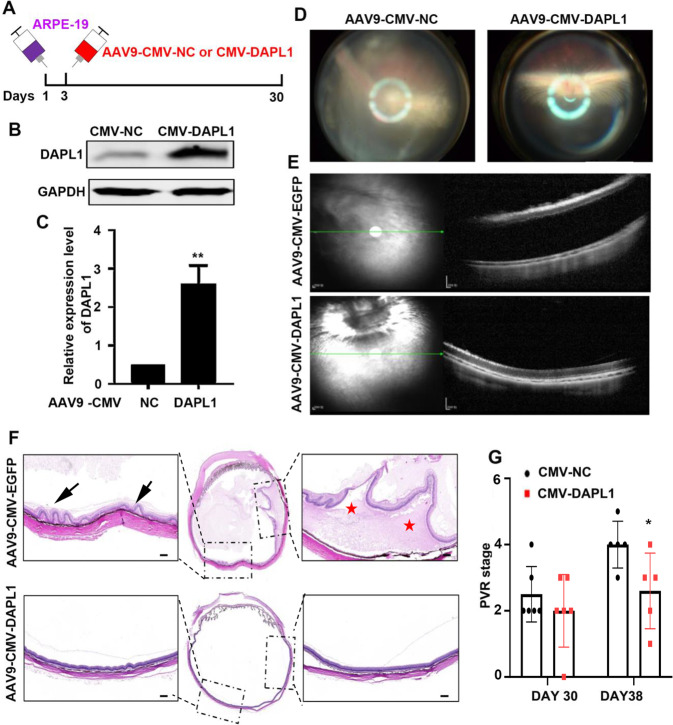
AAV-mediated DAPL1 overexpression prevents the progression of experimental PVR. **A** Schematic diagram of AAV9-CMV-DAPL1. ARPE-19 cells were injected into the vitreous of the Chinchilla rabbits to induce experimental PVR. AAV9-CMV or AAV9-CMV-DAPL1 virus was injected at day 3, and the PVR severity was evaluated at days 30 and 38. **B**, **C** Western blots showing the expression of DAPL1 in ARPE-19 cells after infection with AAV9-CMV-DAPL1 for 2 days and related quantification. *N* = 3. **D** Fundus photographs in each group were captured at day 38. Note that retinal detachment with retinal folds was less severe in the AAV9-CMV-DAPL1-injected eyes. **E** Optical coherence tomography (OCT) scans were performed at day 38, and severe retinal detachment could be observed in the AAV9-CMV-NC-injected eyes, but the AAV9-CMV-DAPL1-injected eyes showed normal retinal structures. **F** Histological images of H&E staining of the rabbit eye in each indicated group showing retinal folds and severe retinal detachment in the AAV9-CMV-NC-injected eyes but a normal retinal structure in the AAV9-CMV-DAPL1-injected eyes. **G** PVR severity in each group was graded according to Fastenberg’s score at the indicated times. *N* = 6, ** indicates *P* < 0.01.

Finally, we addressed whether DAPL1 gene transfer could be used as a therapy for experimental PVR under these disease conditions. To this end, we injected 5 × 10^5^ ARPE-19 cells in a 20 μl volume into the vitreous of the Chinchilla rabbit to induce experimental PVR, and AAV9-CMV or AAV9-CMV-DAPL1 virus was injected at day 3; PVR severity was evaluated at days 30 and 38 (Fig. 8A). Western blot analysis showed that the injection of AAV9-CMV-DAPL1 led to a dramatic increase in the DAPL1 protein level in the infected ARPE-19 cells (Fig. 8B, C). The fundus photographs (Fig. 8D) and OCT (Fig. 8E) analyses showed that AAV9-CMV-NC-injected eyes displayed severe retinal detachment, whereas the AAV9-CMV-DAPL1-injected eyes had a much more organized retinal structure. H&E staining also confirmed the results of retinal detachment (Fig. 8F). Upon evaluating PVR severity according to Fastenberg’s score, the AAV9-CMV-DAPL1-mediated overexpression of DAPL1 efficiently inhibited the progression of PVR (Fig. 8G). These results suggest that gene transfer-mediated DAPL1 overexpression prevents the severity of experimental PVR progression.

## Discussion

In this work our findings reveal that DAPL1 acts as a novel suppressor of RPE-EMT in mice, and that it can antagonize PVR progression in an experimental model. This conclusion is based on multiple observations. First, the expression level of DAPL1 is decreased in the PVR membranes of PVR patients. Second, DAPL1 deficiency promotes RPE-EMT in mice. Third, the overexpression of DAPL1 in RPE cells inhibits the EMT of RPE cells both in vitro and in vivo. Fourth, DAPL1 enhances P21 stabilization partially through NFκB (RelA), and the knockdown of P21 neutralizes the function of DAPL1 in inhibiting RPE-EMT and PVR progression. Fifth, in the experimental PVR state, AAV9-CMV-DAPL1 gene therapy could effectively reduce the severity of experimental PVR progression. Hence, the molecular process involved in this event seems to be arranged linearly in the following way: DAPL1➝NFκB➝ P21 stabilization —| RPE-EMT. This is likely only one of many pathways involved in maintaining the homeostasis of RPE cells and inhibiting EMT in the retina.

The link between DAPL1 and RPE-EMT is of particular interest because it is well established that RPE-EMT is one of the main causative factors in the pathogenesis of PVR [3, 4]. In addition, RPE-EMT also occurs in other retinopathies, including AMD and inherited retinal degeneration [6]. AMD is one of the leading causes of irreversible blinding diseases, and a synonymous single nucleotide polymorphism (rs17810398) in the *DAPL1* gene was reported to be a female-specific susceptibility locus for AMD [16]. Our work demonstrated that *Dapl1*−/− animals display age-related retinal degeneration (unpublished results), but the precise underlying mechanisms are still being studied. EMT is important for the dysfunction of RPE cells, suggesting that DAPL1-mediated regulation of RPE cell EMT might not only be involved in PVR pathogenesis but could also participate in the regulation of other retinopathies, such as AMD.

Our previous study indicated that DAPL1 inhibits RPE cell proliferation by up-regulating P21 protein levels, but its regulatory mechanism remains unknown [20]. Here, we further show that DAPL1 does not regulate the mRNA level of *P21* in ARPE-19 cells, but regulates P21 protein phosphorylation and stabilization. P21 is a cell proliferation inhibitor, which plays critical roles in the regulation of RPE cell proliferation and differentiation [26]. The overexpression of P21 in RPE cells could inhibit PVR progression in a rabbit model [27]. Our current data proved that the knockdown of P21 in DAPL1-overexpressing ARPE-19 cells promotes the EMT of RPE cells, which was based on a decrease in ZO-1, E-cadherin and increase in N-cadherin, vimentin and SNAIL expression. Consistent with our findings, P21 has been reported to regulate the EMT of ovarian cancer and non-small-cell lung cancer cells [33, 34].

For the past several decades, increasing evidence indicates that P21 expression is tightly controlled by multiple mechanisms, including P53-dependent and independent transcriptional regulation and post-transcriptional regulation [35]. The stability of P21 protein is essential for proper cell fate decisions, and this can be regulated by MEK/ERK, AKT, JNK, and P38 pathways [36, 37]. At the post-transcriptional, the phosphorylation event has been reported to increase the stability of P21 [30, 38]. In fact, in the current work we showed that DAPL1 promoted the phosphorylation of P21 protein and increased its stability in RPE cells partially through NFκB (RelA), an important target of the PI3K/AKT pathway [39]. In addition, NFκB could interact with Wnt/β-catenin signaling pathways [40], while GSK-3β, the key mediator of Wnt/β-catenin, was reported to regulate the phosphorylation of P21 [41]. Moreover, NFκB can also directly regulate the transcription of *P21* by binding to its promoter region [42], and its activity also affects heterogonous pathways, such as the P53 axis, an important regulator of P21 [43]. However, the precise mechanisms through which DAPL1 regulates NFκB and then promotes P21 stabilization have not been fully elucidated, and further investigation is needed to address the issue. Moreover, NFκB participates in the regulation of multiple biological processes, including immune responses, inflammatory reactions, and apoptosis [43], which suggests that DAPL1 might have other biological functions in RPE cells and other RPE-dysfunction related retinopathies.

Together, our findings in this study have provided new evidence that in addition to the known functions of DAPL1, it also plays a role in the inhibition of EMT in RPE cells by up-regulating P21 protein stability. The overexpression of DAPL1 in RPE cells can prevent EMT and reduce the severity of experimental PVR progression. As the EMT of RPE cells is closely related to RPE dysfunction and relative retinopathies, we believe that these findings will provide novel insights into the regulatory mechanisms of not only RPE-EMT and PVR but also other forms of retinal diseases in humans, suggesting potential therapeutic interventions for the treatment of RPE dysfunction-related retinopathies in the future.

## Supplementary information

supplement data

Original Data File

Reproducibility checklist

## Data Availability

The datasets generated during and/or analyzed during the current study are available from the corresponding author on reasonable request.
