# Supplementary material for: DAPL1 prevents epithelial–mesenchymal transition in the retinal pigment epithelium and experimental proliferative vitreoretinopathy
Source: Cell Death Dis. 2023 Feb 25;14(2):158. doi: 10.1038/s41419-023-05693-4 (PMC9968328; doi:10.1038/s41419-023-05693-4)
Supplement: Supplementary file 1 — supplement data [file 41419_2023_5693_MOESM1_ESM.pdf]

Supplementary Information for

DAPL1 prevents epithelial–mesenchymal transition in the retinal pigment epithelium and experimental proliferative vitreoretinopathy

Xiaoyin Ma[1,2#*], Shuxian Han[1,3#], Youjia Liu[1#], Yu Chen[1,2], Pingping Li[1], Xiaoyan Liu[1], Lifu Chang[1], Yingao Chen[1], Feng Chen[4], Qiang Hou[2], Ling Hou[1,2*]

# These authors contributed equally to this work.

* Corresponding authors. E-mail address: lhou@eye.ac.cn; xyma2015@wmu.edu.cn

Ma et al., Fig. S1

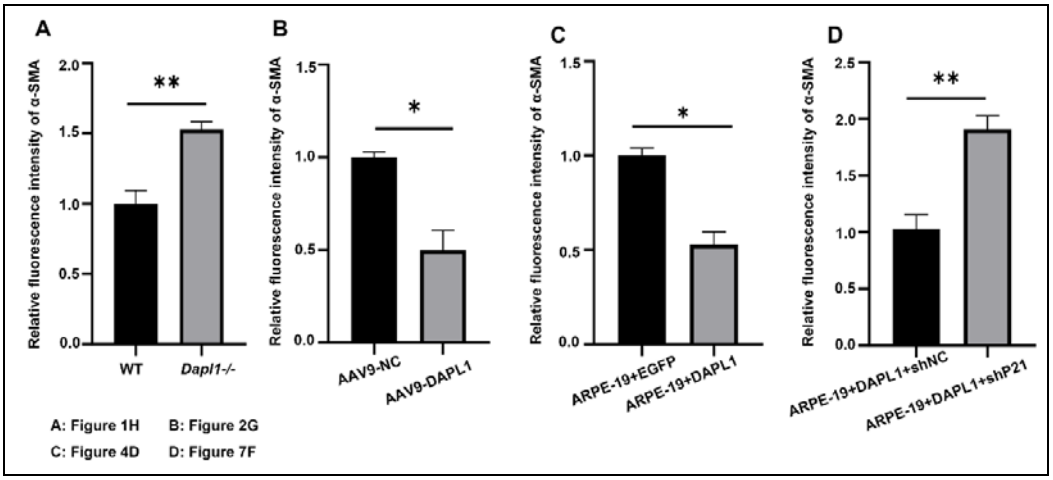

Figure S1: Quantitative analysis of the relative fluorescence intensity of α-SMA. The anti-α-SMA fluorescence in each projected fluorescent image was quantified using Image J. For quantification, the images were color-split, the particle edges were smoothed , the image threshold automatically adjusted and applied. (A-D) Quantitative analysis of the α-SMA fluorescence intensity based on the results of Figure 1H, Figure 2G, Figure 4D and Figure 7F respectively. ** or * indicates $P < 0.01$ or *$P < 0.05$.
