# Supplementary material for: DAPL1 prevents epithelial–mesenchymal transition in the retinal pigment epithelium and experimental proliferative vitreoretinopathy
Source: Cell Death Dis. 2023 Feb 25;14(2):158. doi: 10.1038/s41419-023-05693-4 (PMC9968328; doi:10.1038/s41419-023-05693-4)

Figure1 B

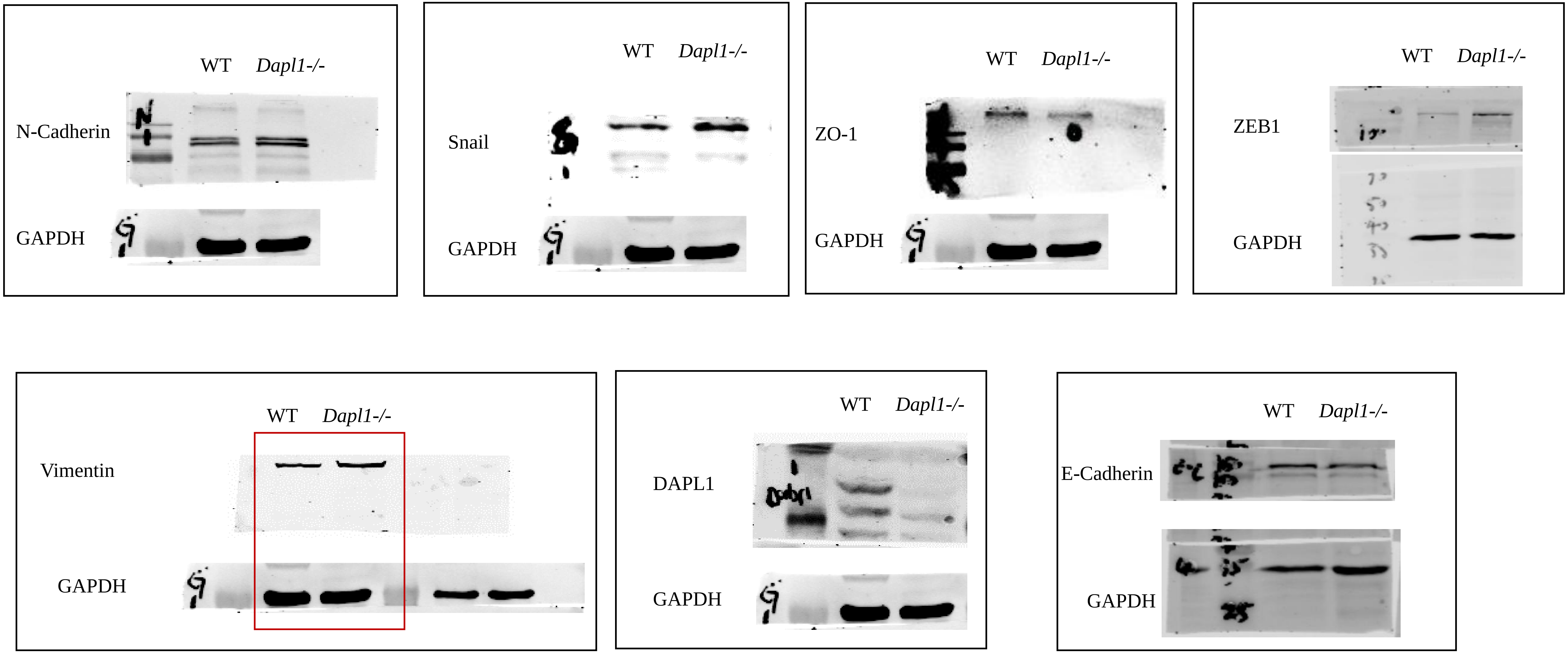

WT
Dapl1-/-
N-Cadherin
GAPDH
WT
Dapl1-/-
Snail
GAPDH
WT
Dapl1-/-
ZO-1
GAPDH
WT
Dapl1-/-
ZEB1
GAPDH
WT
Dapl1-/-
Vimentin
GAPDH
WT
Dapl1-/-
DAPL1
GAPDH
WT
Dapl1-/-
E-Cadherin
GAPDH

Figure2 A

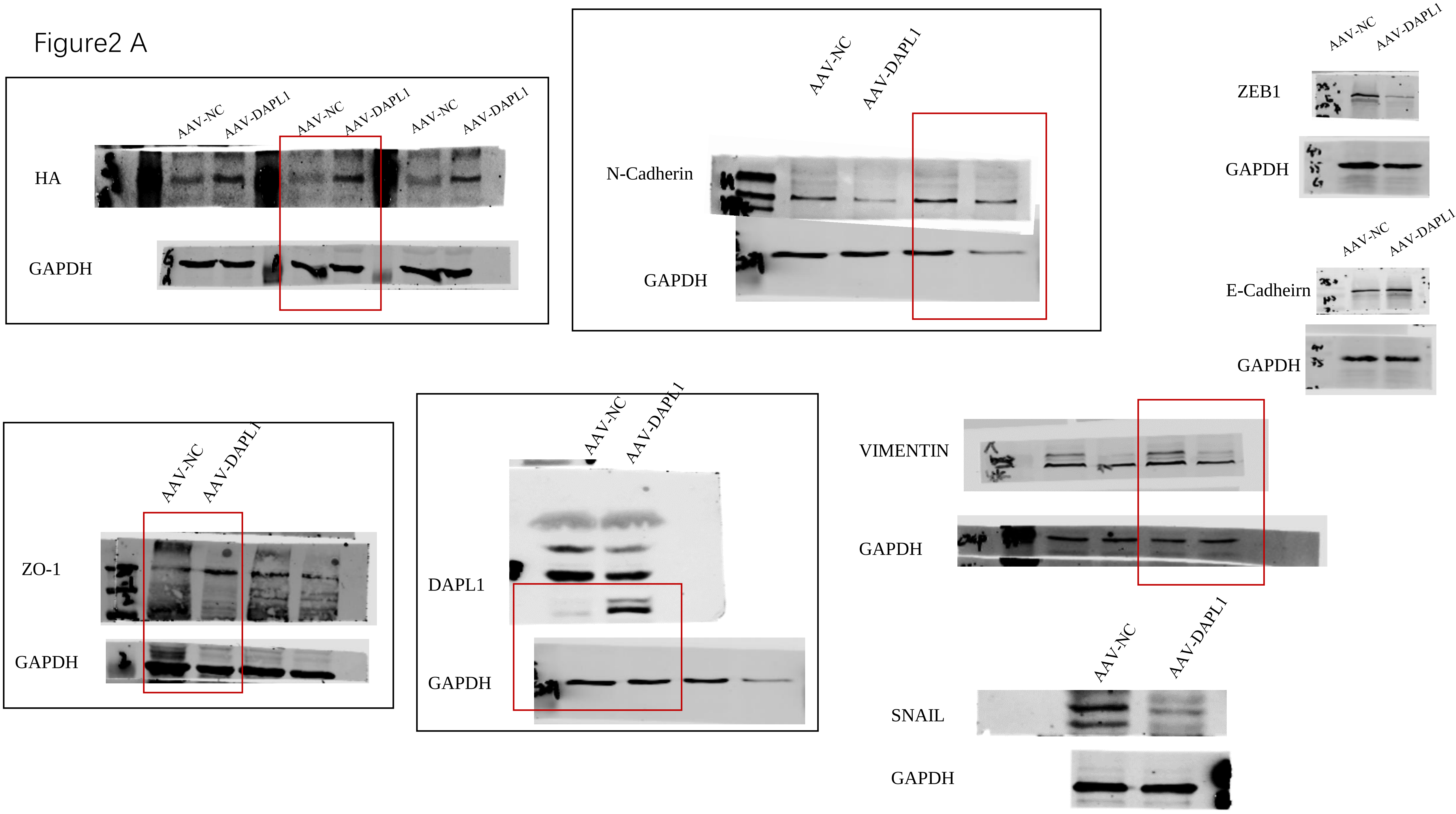
AAV-NC
AAV-DAPL1
HA
GAPDH
N-Cadherin
ZEB1
E-Cadheirn
ZO-1
DAPL1
VIMENTIN
SNAIL

Figure3 A

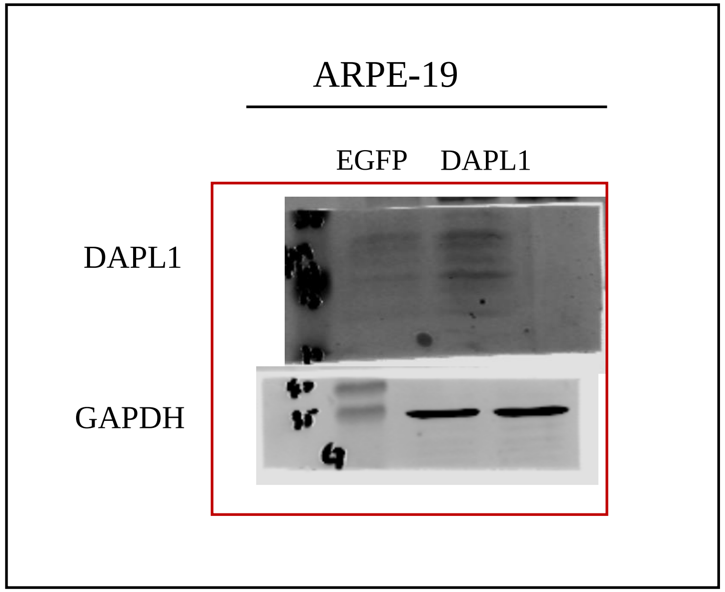

Figure3 G

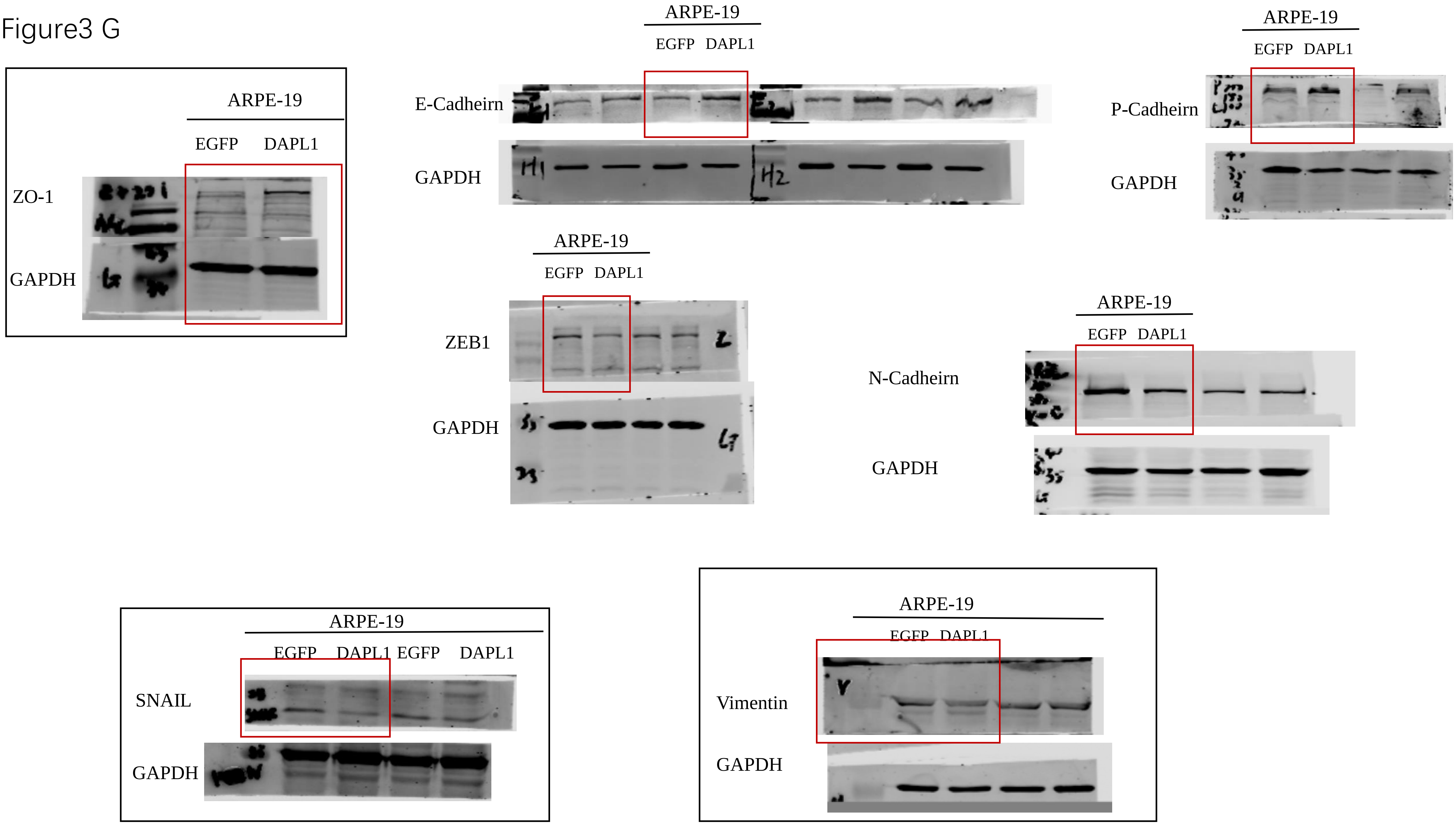

ARPE-19
EGFP
DAPL1
ZO-1
GAPDH
ARPE-19
EGFP DAPL1
E-Cadheirn
GAPDH
ARPE-19
EGFP DAPL1
P-Cadheirn
GAPDH
ARPE-19
EGFP DAPL1
ZEB1
GAPDH
ARPE-19
EGFP DAPL1
N-Cadheirn
GAPDH
ARPE-19
EGFP DAPL1 EGFP DAPL1
SNAIL
GAPDH
ARPE-19
EGFP DAPL1
Vimentin
GAPDH

Figure5 A

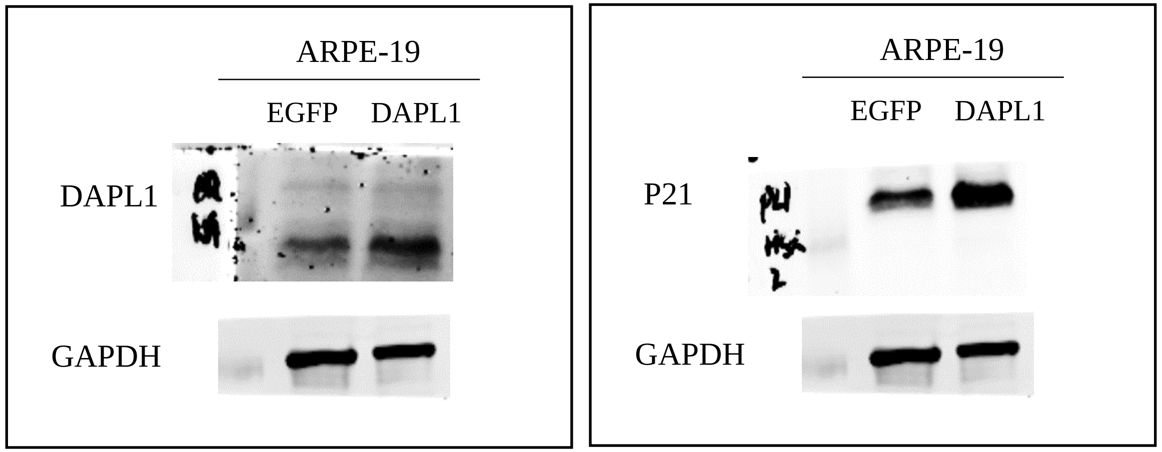

Figure5 C

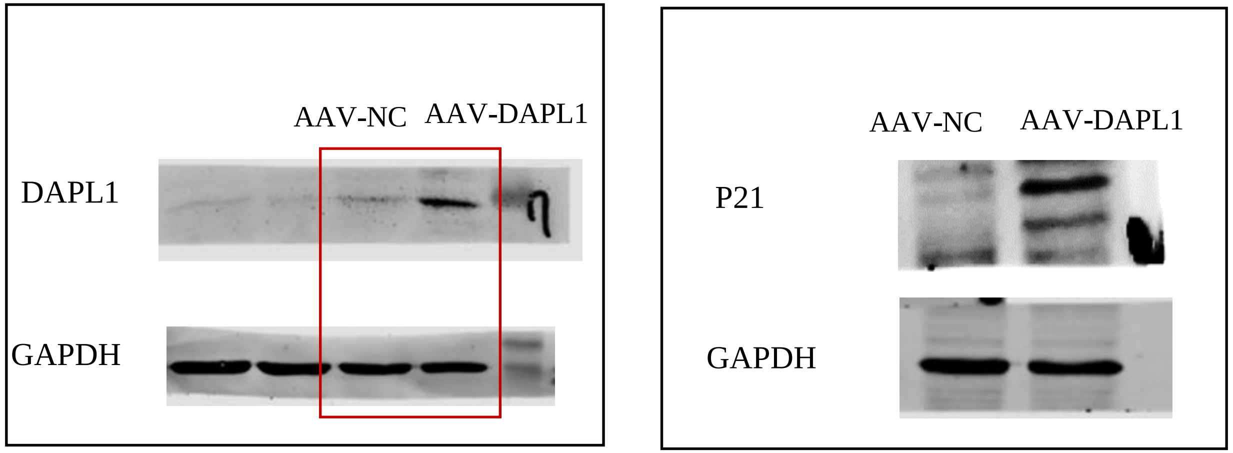

Figure5 F

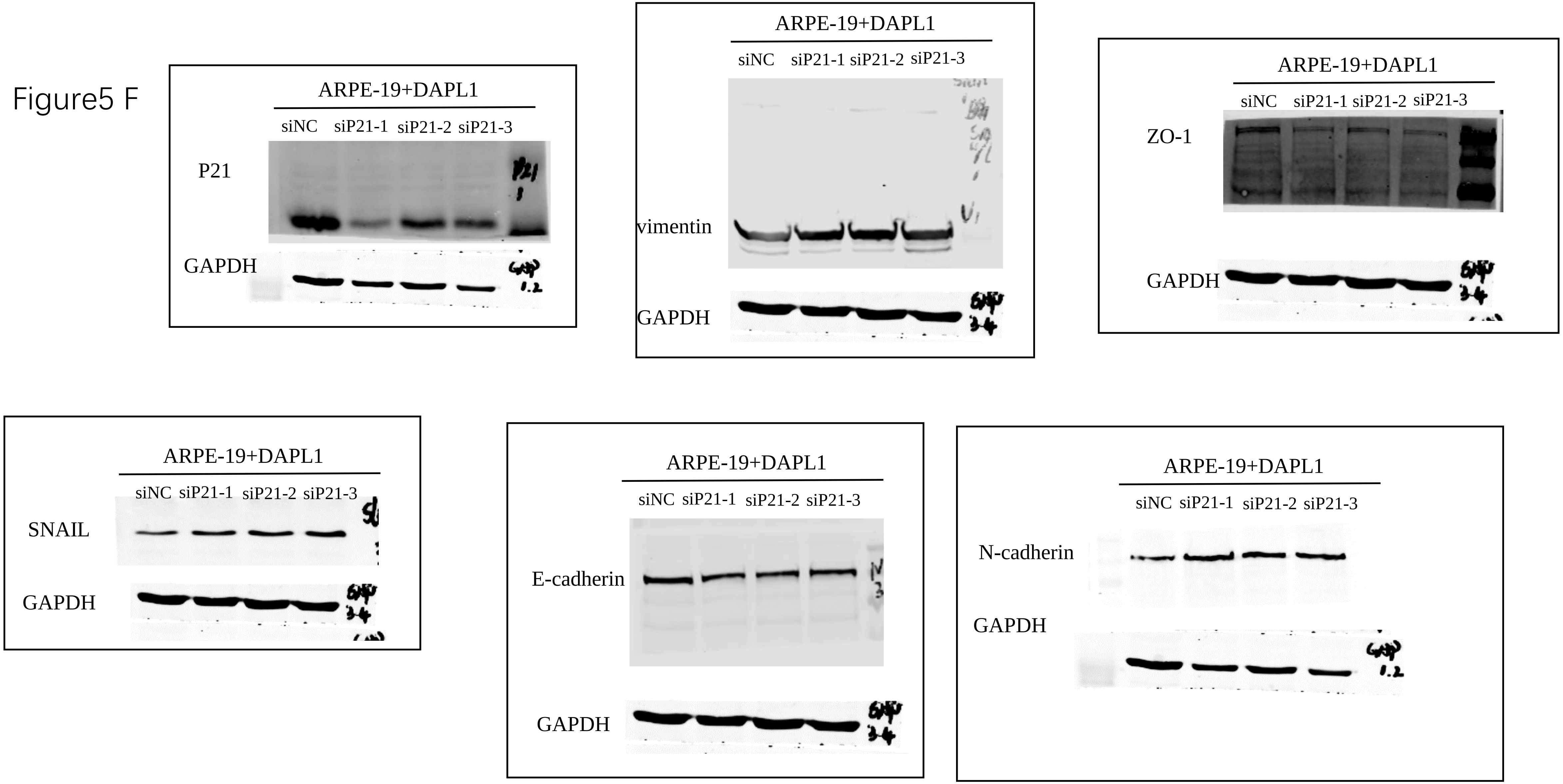
ARPE-19+DAPL1
siNC siP21-1 siP21-2 siP21-3
P21
GAPDH
ARPE-19+DAPL1
siNC siP21-1 siP21-2 siP21-3
vimentin
GAPDH
ARPE-19+DAPL1
siNC siP21-1 siP21-2 siP21-3
ZO-1
GAPDH
ARPE-19+DAPL1
siNC siP21-1 siP21-2 siP21-3
SNAIL
GAPDH
ARPE-19+DAPL1
siNC siP21-1 siP21-2 siP21-3
E-cadherin
GAPDH
ARPE-19+DAPL1
siNC siP21-1 siP21-2 siP21-3
N-cadherin
GAPDH

Figure6 B

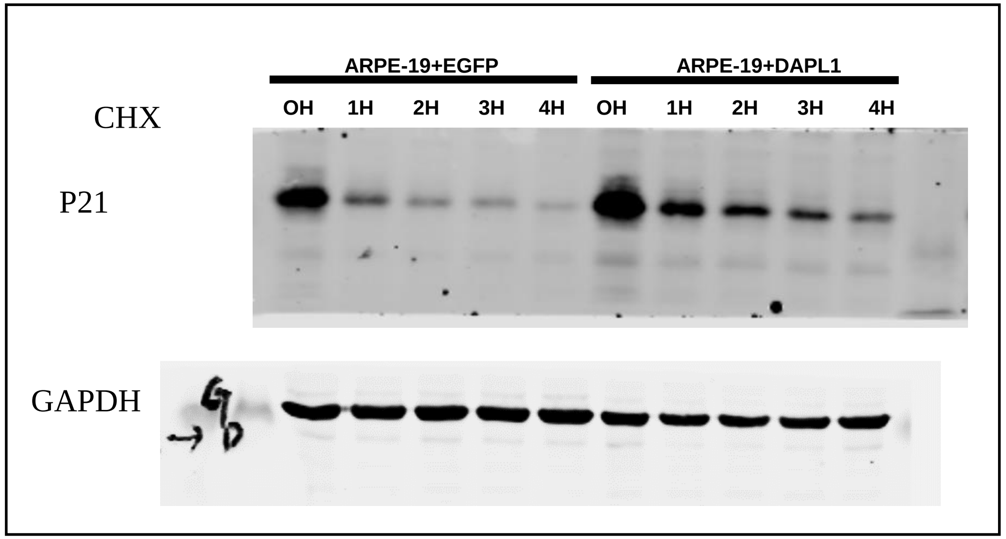

Figure6 D

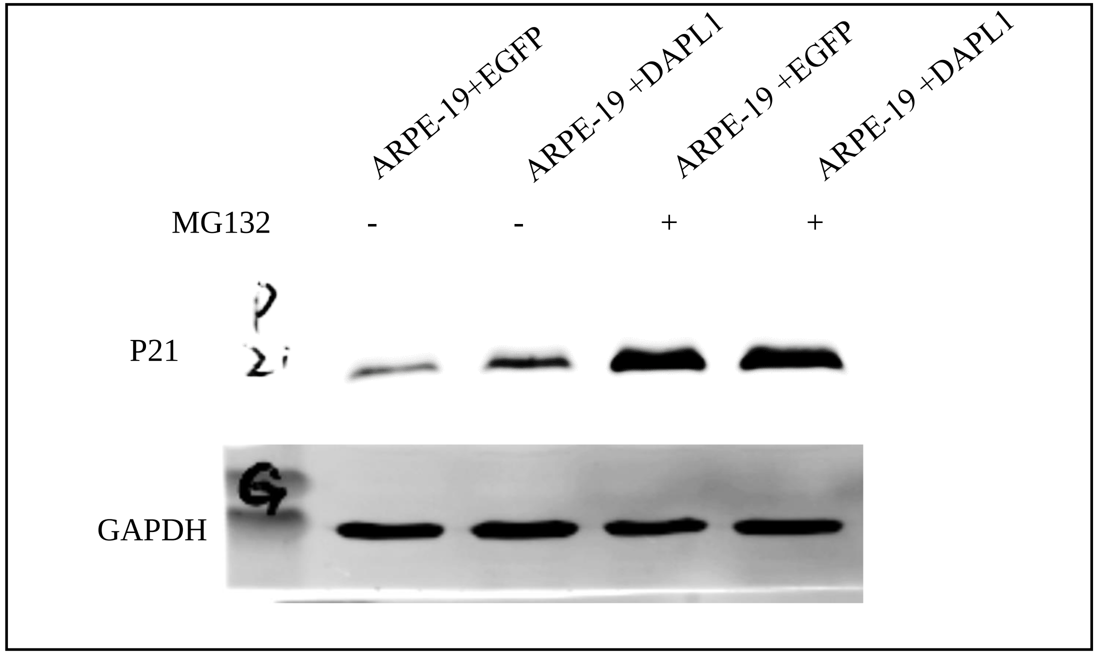

Figure6 F

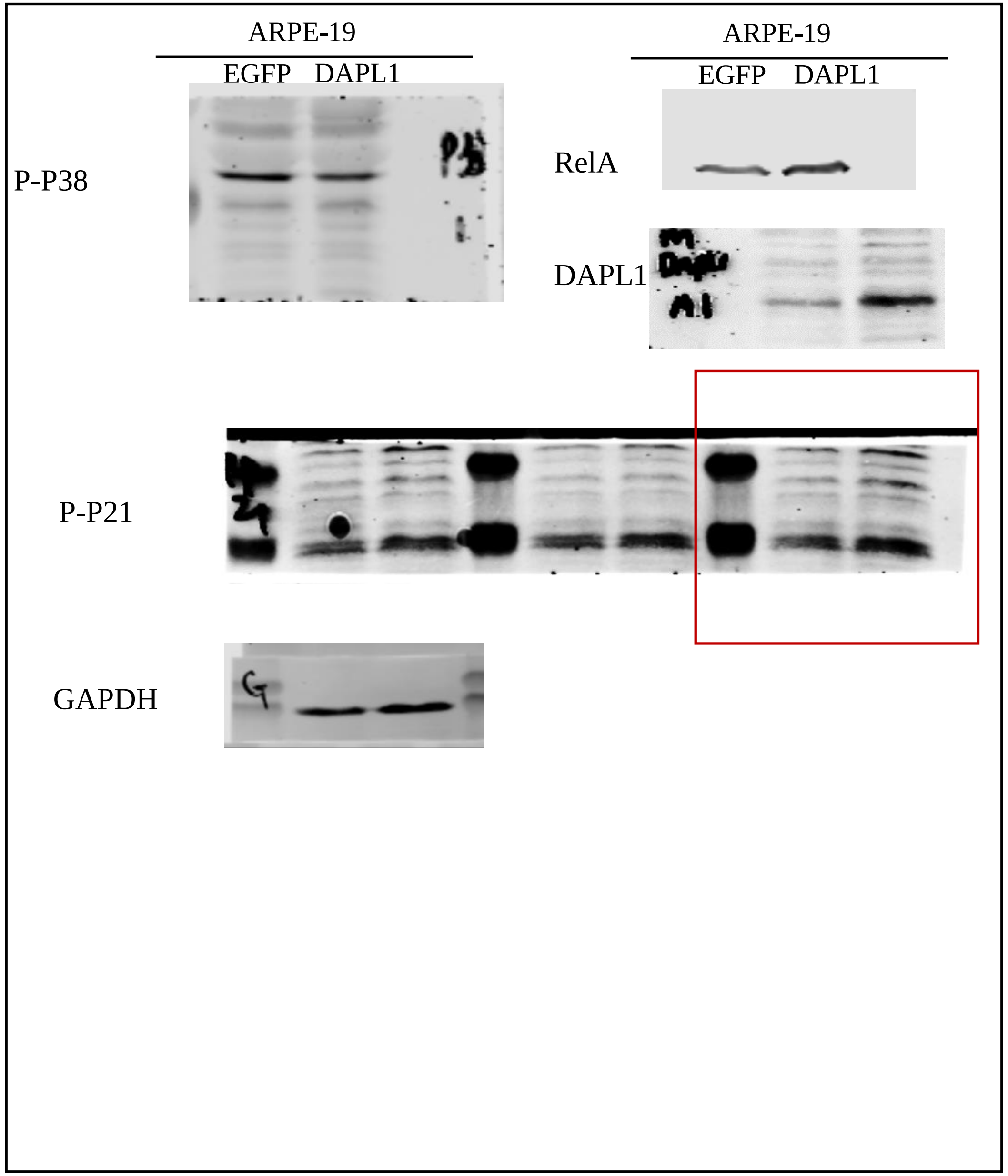

Figure6 H

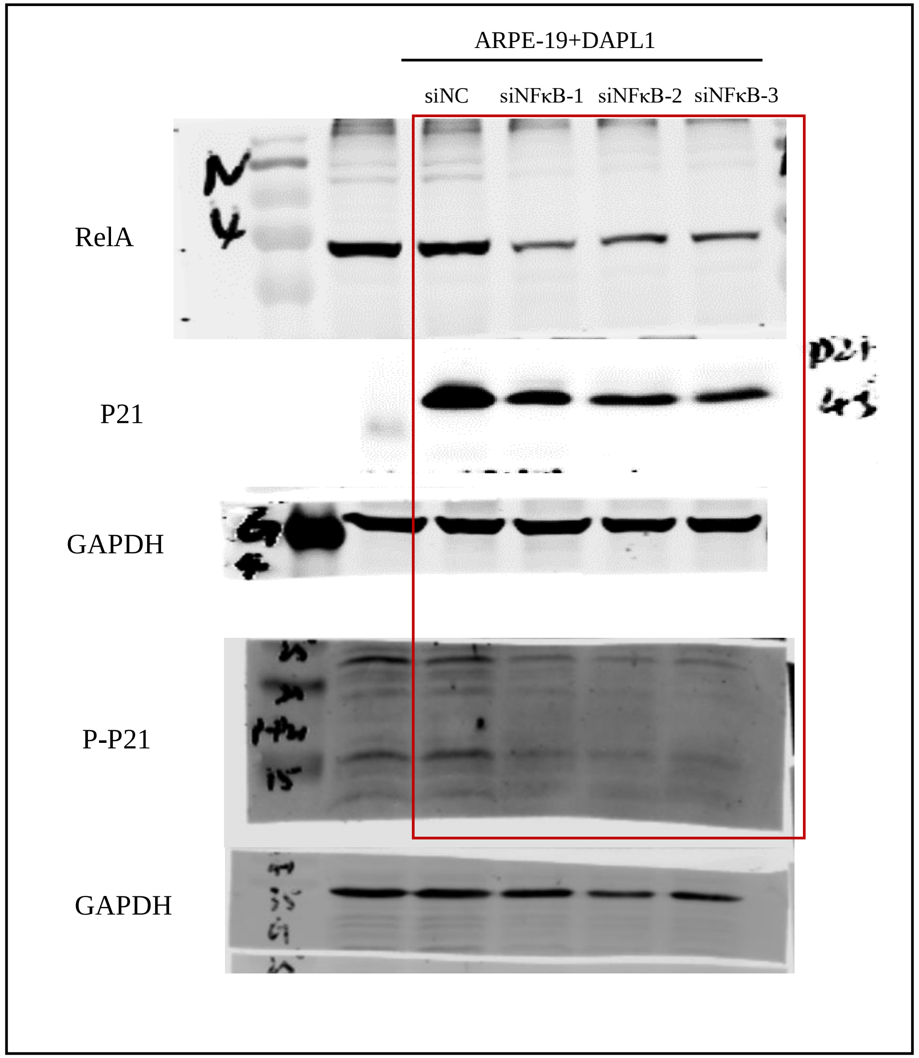

Figure6 J

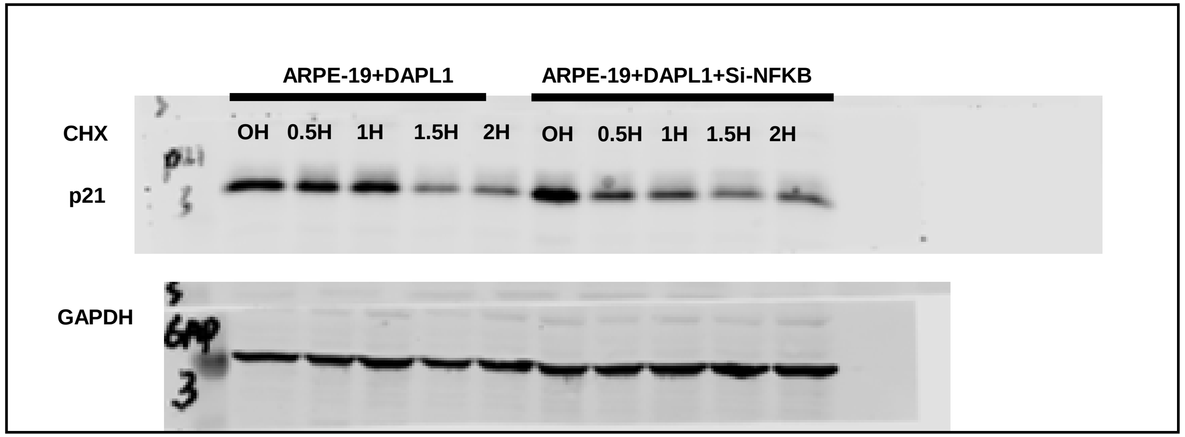

Figure7A

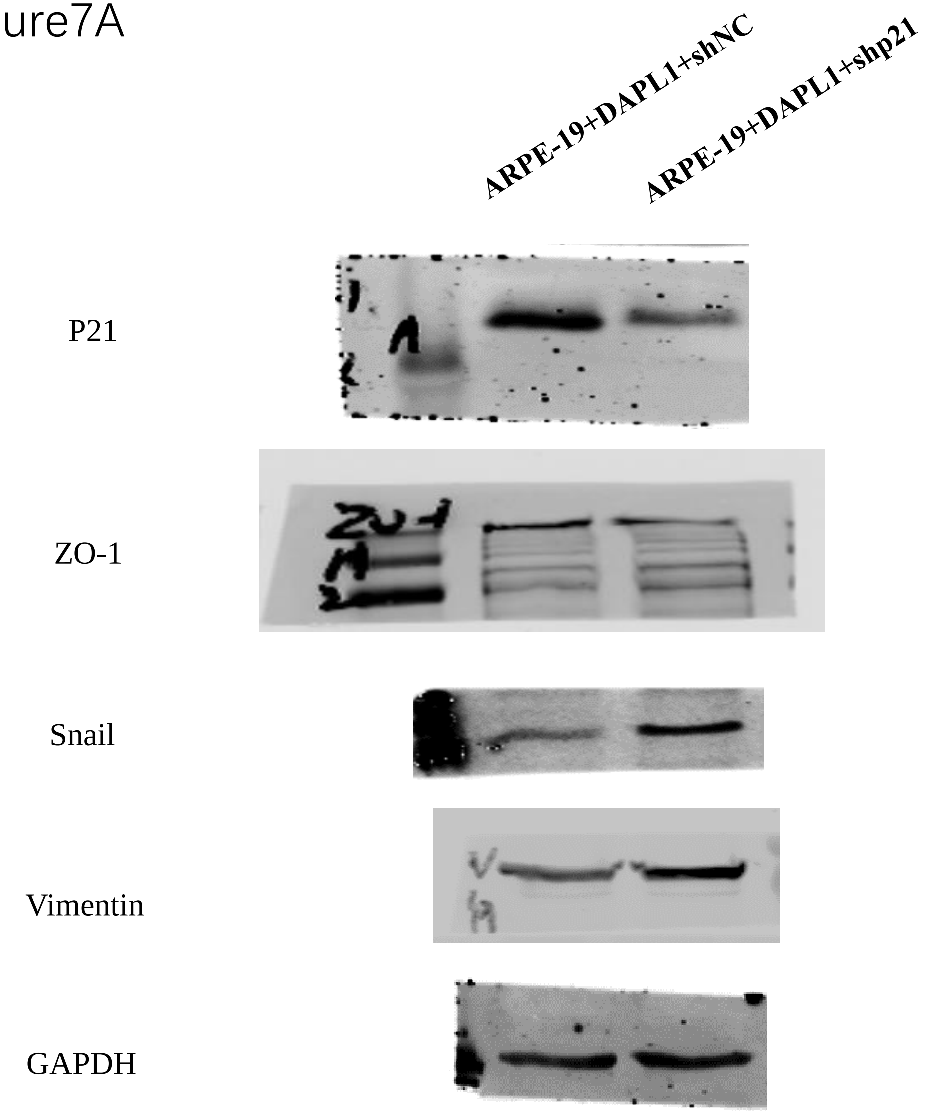

Figure8 B

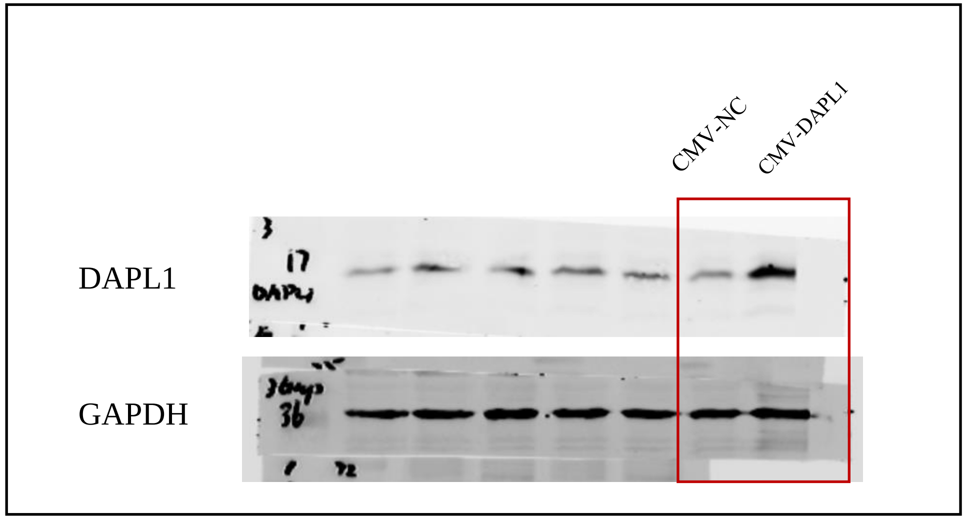

Supplement: Supplementary file 2 — Original Data File [file 41419_2023_5693_MOESM2_ESM.pdf]
